# Evaluation of the *in vitro* susceptibility of clinical isolates of NDM-producing *Klebsiella pneumoniae* to new antibiotics included in a treatment regimen for infections

**DOI:** 10.3389/fmicb.2024.1331628

**Published:** 2024-04-05

**Authors:** Natalia Słabisz, Patrycja Leśnik, Jarosław Janc, Miłosz Fidut, Marzenna Bartoszewicz, Ruth Dudek-Wicher, Urszula Nawrot

**Affiliations:** ^1^Department of Laboratory Diagnostic, 4th Military Clinical Hospital, Wroclaw, Poland; ^2^Department of Microbiology, Wroclaw Medical University, Wroclaw, Poland; ^3^Department of Anaesthesiology and Intensive Therapy, Hospital of Ministry of the Interior and Administration, Wroclaw, Poland; ^4^Department of Cardiology, 4th Military Clinical Hospital, Wroclaw, Poland; ^5^Department of Pharmaceutical Microbiology and Parasitology, Wroclaw Medical University, Wroclaw, Poland

**Keywords:** *Klebsiella pneumoniae*, metallo-beta-lactamase, NDM, susceptibility, aztreonam, eravacycline, fosfomycin, tigecycline

## Abstract

**Background:**

Due to the growing resistance to routinely used antibiotics, the search for new antibiotics or their combinations with effective inhibitors against multidrug-resistant microorganisms is ongoing. In our study, we assessed the *in vitro* drug susceptibility of *Klebsiella pneumoniae* strains producing New Delhi metallo-β-lactamases (NDM) to antibiotics included in the Infectious Diseases Society of America (IDSA) and European Society of Clinical Microbiology and Infectious Diseases (ESCMID) recommendations.

**Methods:**

A total of 60 strains of NDM-producing *K. pneumoniae* were obtained from different patients hospitalized at the 4th Military Hospital in Wroclaw between 2019 and 2022 and subjected to drug susceptibility to selected antibiotics, including the effects of drug combinations.

**Results:**

Among the tested antibiotics, the highest sensitivity (100%) was observed for cefiderocol, eravacycline (interpreted according to the European Committee on Antimicrobial Susceptibility Testing [EUCAST]), and tigecycline. Sensitivity to intravenous fosfomycin varied depending on the method used. Using the “strip stacking” method, determining cumulative sensitivity to ceftazidime/avibactam and aztreonam demonstrated 100% *in vitro* sensitivity to this combination among the tested strains.

**Conclusion:**

The *in vitro* susceptibility assessment demonstrated that, the best therapeutic option for treating infections caused by carbapenemase-producing strains seems to be a combination of ceftazidime/avibactam with aztreonam. Due to the safety of using both drugs, cost effectiveness, and the broadest indications for use among the tested antibiotics, this therapy should be the first-line treatment for carbapenemase-producing *Enterobacterales* infections. Nevertheless, a comprehensive evaluation of the efficacy of treating infections caused by NDM-producing *K. pneumoniae* strains should include not only *in vitro* susceptibility assessment but also an analysis of clinical cases.

## 1 Introduction

Recently, a rapid spread of carbapenemase-producing *Enterobacterales* (CPE) strains has been observed worldwide, including in Poland, which represents a severe epidemiological and therapeutic problem. CPE most often colonizes the gastrointestinal tract but can also cause urinary tract infections (UTIs), pneumonia, or blood stream infection. Antimicrobial resistance genes are included in mobile elements such as plasmids, transposons, and integrons. The importance of these elements lies in their role in the vertical transmission of genes from *Klebsiella pneumoniae* to its descendants, as well as the horizontal transmission of genes from a specific *K. pneumoniae* strain to another (Karampatakis et al., 2023). These microorganisms spread very quickly in the hospital environment, primarily through direct contact with another patient who is a carrier of CPE or through the hands of medical staff. Screening of patients from risk groups during admission to the hospital, adherence to hand hygiene procedures by medical staff, and the application of rational antibiotic therapy in healthcare units, constitute the primary tool in the fight against spreading of CPE infections (Otter et al., 2020).

Most CPE strains are completely resistance to commonly used antibiotics, that is why, treating infections caused by these microorganisms often requires new, unconventional antibiotics or combination antibiotic therapy based on two or even three drugs. Unfortunately, in the case of CPE strains, there are often only one or two therapeutic options left for treatment, and there are also situations in which the strain is entirely resistant to all known antibiotics. Therefore, both laboratories and clinicians are forced to look for combinations of “old” and “new” antibiotics, the combined action of which may provide a chance for therapeutic success (Karaiskos et al., 2019; Ontong et al., 2021). Recently registered new antibiotics such as plazomicin, eravacycline or cefiderocol, may be an effective remedy in the fight against infections caused by CPE strains (Castanheira et al., 2020; McCreary et al., 2021; Zou et al., 2023).

In 2021, the European Society of Clinical Microbiology and Infection Diseases (ESCMID) published recommendations containing proposed treatment regimens for infections caused by third-generation cephalosporins- resistant microorganisms and *Enterobacterales, Pseudomonas aeruginosa, Acinetobacter baumannii* that are resistance to carbapenems (Paul et al., 2022).

In the case of enterobacterial rod-producing metallo-β-lactamases, in patient with severe infections, it is recommended to use cefiderocol or combination of aztreonam with ceftazidim/avibactam. In particular, the synergistic effects of a variety of aztreonam combined with ceftazidime/avibactam deserves attention. Further, the sensitivity of CRE-MBL to old antibiotics, including polymyxins, tigecycline, or fosfomycin IV, has been reported. In each of these cases, the drug susceptibility of the individual strains should be determined. In 2021 and 2023, similar recommendations were made by the Infectious Diseases Society of America (IDSA; Tamma et al., 2023).

The minimum inhibitory concentration (MIC) method is commonly employed in microbiological diagnostics to determine the lowest concentration of an antimicrobial agent that effectively inhibits the growth of a specific microorganism. There are also more specialized diagnostic tools to assess the interaction of two different antibiotics. This effect may be synergistic, additive, neutral, or antagonistic. Choosing this reciprocal relationship between the two drugs is a crucial therapeutic clue in treating infections caused by carbapenemase-producing *Enterobacterales* (Massoni-Cristante et al., 2003; Avery and Nicolau, 2018; Maraki et al., 2021; Terbtothakun et al., 2021).

Among carbapenemases such as IMP (active against imipenem; imipenemase), VIM (Verona integron-encoded metallo-β-lactamase), KPC (*K. pneumoniae* carbapenemase), New Delhi metallo-β-lactamases (NDM), and OXA-48-like, NDM constitutes a critical medical issue. The effectiveness of almost all *β*-lactams, including carbapenems, is compromised by this enzyme, except for aztreonam and cefiderocol. Given that, there are very few antibiotics available as therapeutic options. The objective of this study was to assess the susceptibility of clinical isolates of NDM-producing *K. pneumoniae*, recognized as a significant threat to public health and a common factor in nosocomial infections, to new antibiotics, including drugs recommended by U.S. Food and Drug Administration (2023), IDSA, and ESCMID for treatment of nosocomial and complicated infections, presented in Table 1.

**Table 1:** Antibiotics recommended by FDA, IDSA, and ESCMID for the treatment of CPE infections (U.S. Food and Drug Administration, 2018; Paul et al., 2022; Tamma et al., 2023).

Antibiotics	Mechanism of action	Indications^a^
Cefiderocol (Fetcroja®) (siderophore cephalosporin)	Cefiderocol binds to extracellular free iron via its siderophore side chain, allowing active transport into the periplasmic space of Gram-negative bacteria through siderophore uptake systems. subsequently binds to penicillin-binding proteins (PBPs), inhibiting bacterial peptidoglycan cell wall synthesis, which leads to cell lysis and death.	cUTI caused by susceptible strains of *E. coli, K. pneumoniae, P. mirabilis, P. aeruginosa, E. cloacae* HAP, VAP caused by *A. baumanii* complex*, E. coli, E. cloacae* complex, *K. pneumoniae*, *P. aeruginosa*, *S. marcescens* Bacteriemia Should be used to treat patients who have limited treatment options only after consultation with a physician with appropriate experience in the management of infectious diseases.
Eravacyclin (Xerava®) (synthetic fluocycline tetracycline)	The mechanism of action of eravacycline involves the disruption of bacterial protein synthesis by binding to the 30S ribosomal subunit thus preventing the incorporation of amino acid residues into elongating peptide chains.	cIAI caused by *E. coli, K. pneumoniae, E. faecalis, E. faecium, S. aureus, Viridans streptococcus* spp.
Plazomicin (Zemdri®) (semisynthetic aminoglycoside derived from sisomicin)	plazomicin binds to the 16S rRNA at the aminoacyl-tRNA site (A-site) of the 30S ribosomal subunit, interfering with protein translation.	cUTI caused by *E. coli, K. pneumoniae, P. mirabilis, E. cloacae* Active against *Enterobacterales* resistant to β-lactams and other classes of antibacterials May cause nephrotoxicity, ototoxicity and neuromuscular blockade.
Aztreonam (Cayston®) (monobactam β-lactam)	Aztreonam is a bactericidal agent that acts by inhibition of bacterial cell wall synthesis.	UTI (complicated and uncomplicated) Cystic fibrosis Lower respiratory tract infections (pneumoniae, bronchitis) Skin infections IAI Gynecologic infections Systematic infection: severe or life-threatening.
Ceftazidim/avibactam (Zavicefta®) (cephalosporin combined with non-β-lactam β-lactamase inhibitor)	The bactericidal action of ceftazidime is mediated through binding to essential penicillin-binding proteins (PBPs). Avibactam is a non- β lactam β-lactamase inhibitor that inactivates some β-lactamases and protects ceftazidime from degradation by certain β-lactamases.	cUTI cIAI HAP VAP
Fosfomycin IV (InfectoFos®) (phosphonic acid)	Fosfomycin IV inhibits the enzyme phosphoenolpyruvate transferase, which catalyzes the formation of n-acetylmuramic acid from n-acetyl aminoglucose and phosphoenolpyruvate. N-acetylmuramic acid is required for the buildup of peptidoglycan, an essential component of the bacterial cell wall.	cUTI Endocarditis^b^ HAP, VAP^b^ cSSI^b^ Osteomyelitis^b^ cIAI^b^ meningitis^b^
Tigecycline (derivative of Minocycline)	Tigecycline, a glycylcycline, inhibits protein translation in bacteria by binding to the 30S ribosomal subunit and blocking entry of amino-acyl tRNA molecules into the A site of the ribosome. This prevents the incorporation of amino acid residues into elongating peptide chains.	cSSI cIAI CAP

^a^FDA, approved; ^b^EMA, approved (available in Europe, Australia and Canada). cUTI, complicated urinary tract infection; HAP, hospital-acquired pneumonia; VAP, ventilatory-associated pneumonia; cIAI, complicated intra-abdominal infection; UTI, urinary tract infection; IAI, complicated intra-abdominal infection; cSSI, complicated surgical site infection; CAP, community-acquired pneumonia.

The study aimed to evaluate the sensitivity of *K. pneumoniae* NDM isolates obtained from patients with UTI, VAP, and BSI infections in the 4th Military Hospital of Wroclaw from 2019 to 2022 to new antibiotics included in the IDSA and ESCMID recommendations.

## 2 Methods

The study was carried out in the Microbiology Laboratory of the Laboratory Diagnostics Department of the 4th Military Clinical Hospital in Wroclaw, based on material obtained in the Clinical Department of Anesthesiology and Intensive Care and other departments. *K. pneumoniae* NDM strains were obtained from patients’ cultures of clinical materials collected for routine microbiological tests, which were subjected to drug susceptibility to selected antibiotics, including the effects of drug combinations.

### 2.1 Ethics

The study protocol was approved by the Bioethics Committee of the Lower Silesian Medical Chamber, Poland (approval no. 2/BNR/2023). Confidentiality and privacy were considered with regard to personal, laboratory, and clinical data. The study was carried out in accordance with the guidelines of the Declaration of Helsinki and Good Clinical Practice. Written informed consent was obtained from all participants prior to the study.

### 2.2 Microorganisms

A total of 60 strains of NDM-producing *K. pneumoniae* were obtained from different patients hospitalized in the 4th Military Hospital in Wroclaw between 2019 and 2022 and used for the study: 20 strains originating from bloodstream infections, 20 strains isolated from urinary tract infections, and 20 strains from lower respiratory tract specimens (BAL- bronchoalveolar lavage, tracheal aspirates). The bacterial strains all originated from different patients. In the case of infection with the same bacterial strain across multiple systems, only a single isolate was used for testing.

### 2.3 Identification

All *K. pneumoniae* strains were identified using the VITEK MS system (bioMérieux, France), according to the manufacturer’s instructions. The confidence interval for identification of all *K. pneumoniae* strains was 99.9%. *Escherichia coli* ATCC 8739 was used as the quality control strain.

### 2.4 Carbapenemase detection

Phenotypic detection of carbapenemases was performed using the immunochromatography test RESIST-5 O.O.K.N.V (CorisBioConcept, Belgium). This kit aims to detect and identify carbapenemases from a bacterial colony. Lateral-flow tests are based on membrane technology with colloidal gold nanoparticles. Quality control of this method was performed using the reference strain *E. coli* ATCC 25922.

### 2.5 Susceptibility testing

#### 2.5.1 Gradient strip-based method

MIC Test Strip MTS™ (Liofilchem, Italy) is a quantitative method for *in vitro* susceptibility testing. MIC is the minimum inhibitory concentration of an antibiotic that inhibits the growth of bacteria under standardized *in vitro* conditions. MTS™ consists of special porous paper impregnated with a pre-defined concentration gradient of an antimicrobial agent, used to determine the minimum inhibitory concentration (MIC) in μg/mL of antimicrobial agents against bacteria. MTS™ strip tests were performed on Mueller Hinton Agar (bioMérieux, France). Liofilchem™ MTS™ Fosfomycin includes glucose-6-phosphate. The results were read after 16–20 h incubation at 35°C in ambient air. *Escherichia coli* ATCC 25922 was used as the quality control strain.

#### 2.5.2 Agar dilution method (reference method)

Agar dilution is considered the best method for fosfomycin susceptibility testing, as recommended by CLSI and EUCAST standards. AD Fosfomycin 0.25–256 (Liofilchem, Italy) is a 12-well panel containing the antibiotic incorporated into an agar medium in different concentrations (11 two-fold dilutions, growth control). The tested microorganism was first isolated on a suitable non-selective culture medium- Columbia Agar (bioMérieux, France). The standardized suspension of a density of 0.5 McFarland was subsequently diluted 1:10 in saline, and 2 μL of the inoculum solution was inoculated into each well (final inoculum concentration should be approximately 10^4^ CFU per spot). Test setups were incubated at 35°C for 16–20 h in ambient air. According to EUCAST, the MIC was recorded at the minimum concentration where there was non-confluent growth. Single colonies, pinpoint colonies, and a thin film of growth were ignored. Quality control of AD Fosfomycin 0.25–256 (Liofilchem, Italy) was performed using the *E. coli* ATCC 25922 strain (Croughs et al., 2022).

#### 2.5.3 Gradient strip-stacking method

Susceptibility testing of the aztreonam plus ceftazidime/avibactam combination was performed on MH agar (bioMérieux, France) using the MIC Test Strip MTS™ (Liofilchem, Italy). Aztreonam (AZT) strips were placed on culture-inoculated agar surfaces and allowed to diffuse for 10 min. After 10 min, the aztreonam strips were removed, and the ceftazidime/aztreonam (CAZ/AVI) strips were placed at the same location. The aztreonam strips were then placed over the ceftazidime/avibactam strips to help read the MIC values of aztreonam after 16–18 h of incubation in ambient air (Khan et al., 2021; Bakthavatchalam et al., 2022). The cumulative MIC was interpreted against the Clinical and Laboratory Standards Institute (CLSI) criteria for aztreonam (Clinical and Laboratory Standards Institute, 2018).

#### 2.5.4 Interpretation of the results

MIC breakpoints for selected antibiotics according to the European Committee on Antimicrobial Susceptibility Testing (EUCAST), Clinical and Laboratory Standards Institute (CLSI) and FDA are shown in Table 2.

**Table 2:** MIC breakpoints for selected antibiotics according to EUCAST, CLSI and FDA (Clinical and Laboratory Standards Institute, 2018; European Committee on Antimicrobial Susceptibility Testing, 2023; U.S. Food and Drug Administration, 2023).

Antibiotic	Interpretative criteria	MIC breakpoints (mg/L)	
		S≤	R>
Cefiderocol (CDR)	EUCAST	2	2
Eravacycline (ERV)	EUCAST (ECOFF)	2	2
	FDA	0.5	0.5
Tigecycline (TIG)	EUCAST (ECOFF)	2	2
	FDA	2	8 (≥)
Plazomycin (PLZ)	CLSI	2	8 (≥)
Fosfomycin iv (FOS)	EUCAST	32	32
Ceftazidime/avibactam (CAZ/AVI)	EUCAST	8	8
Aztreonam (AZT)	EUCAST	1	4
Ceftazidime/avibactam + aztreonam (CAZ/AVI + AZT)	CLSI	4	16

#### 2.5.5 Quality control

*Escherichia coli* ATCC 25922 strain was used for gradient strip-based method quality control according to the recommendations of EUCAST.

#### 2.5.6 Statistics

In this study, the statistical analyses were conducted using the STATISTICA 13 (TIBCO Software Inc. Palo Alto, United States) software package. To assess the normality of the data distribution, the Shapiro–Wilk test was employed. For the comparison between different groups, the non-parametric, Kruskal–Wallis test was used (with Dunn’s *post-hoc* test). Additionally, to compare the results of FOS (MTS™) with FOS (AD FOSF®) as the reference method, the Mann–Whitney *U*-test was applied. In all statistical tests, a *p* < 0.05 was considered to indicate statistical significance.

## 3 Results

**Figure 1:**
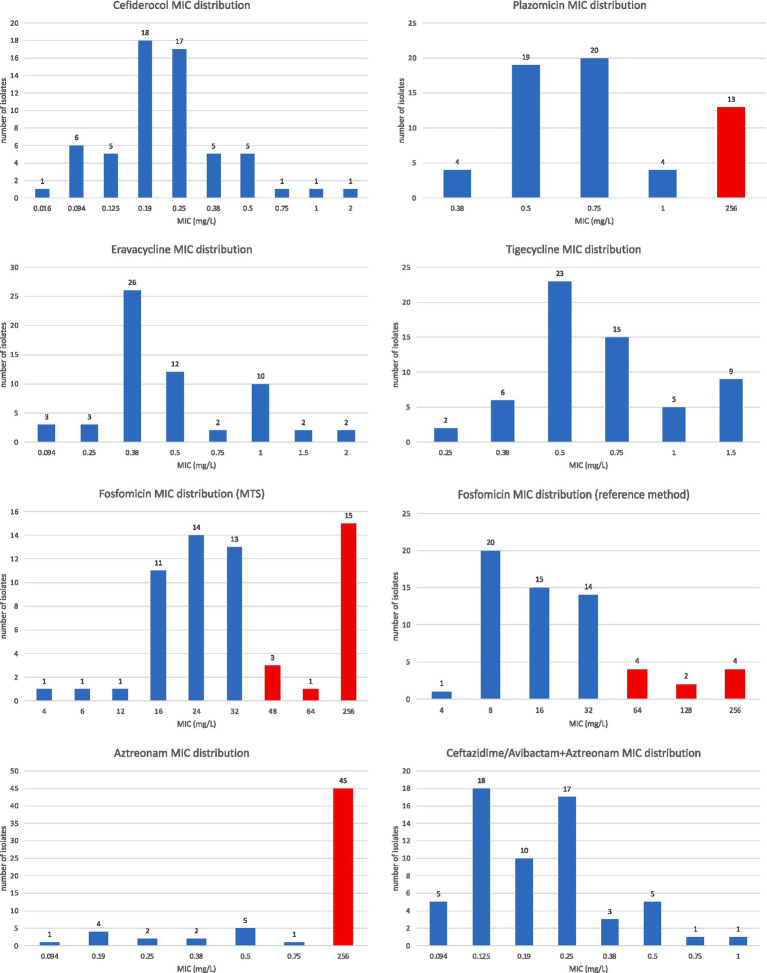
Distribution of MIC values for tested antibiotics. MIC values marked in red are within the resistance category of a given antibiotic.

Among the tested antibiotics, the highest sensitivity (100%) was observed for cefiderocol, eravacycline (interpreted according to EUCAST), and tigecycline. Only 78% of tested strains were susceptibility to plazomycin. Two methods for determining susceptibility to fosfomycin were used in this study. For the gradient strip-based method, susceptibility of tested strains was 68%, compared to 83% using the commercial test AD Fosfomycin 0.25–256 (Liofilchem, Italy) in which the reference method was employed (Figure 1).

**Figure 2:**
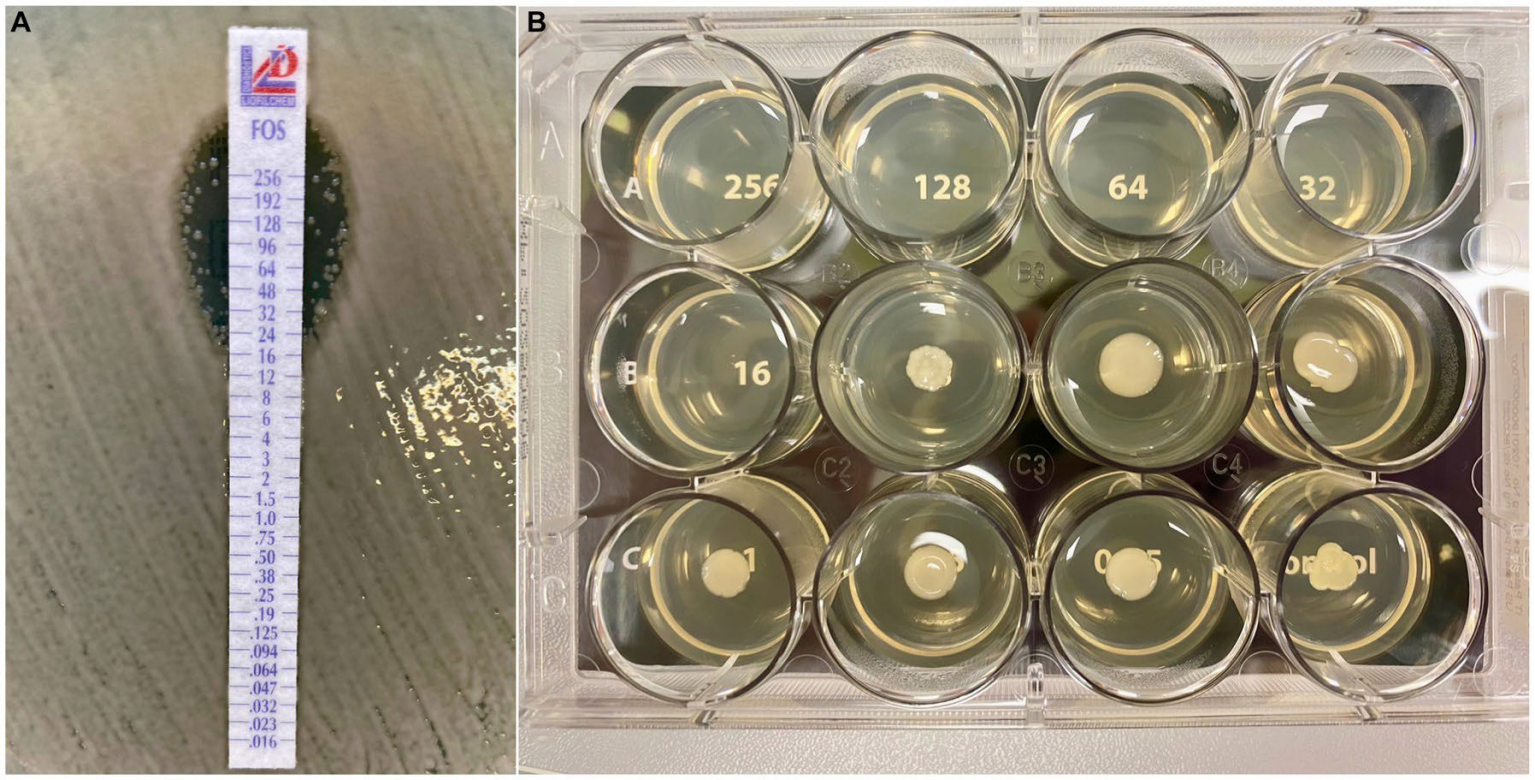
A comparison of susceptibility testing for fosfomycin with **(A)** gradient-strip based method (MIC Test Strip MTS™, Liofilchem, Italy) and with **(B)** reference agar dilution method (AD Fosfomycin 0.25–256, Liofilchem, Italy). In the case of the reference method, the interpretation of the MIC value for the tested strain is straightforward (MIC = 16), whereas the presence of micro and macro colonies within the inhibition zone, when using a gradient strip method, can pose challenges in determining the correct MIC value.

The studied NDM-producing *K. pneumoniae* strains showed 100% resistance to ceftazidime with avibactam and 92% resistance to aztreonam when these drugs were tested individually (Table 3; Figure 2; Supplementary Table S1). Using the “strip stacking” method to determine the cumulative sensitivity to ceftazidime/avibactam and aztreonam demonstrated 100% *in vitro* sensitivity to this combination among the tested strains (Figure 2).

**Table 3:** Number of susceptible isolates, MIC range, MIC 50, MIC 90 (μg/ml) values of the tested antibiotics.

Antibiotic	No of sensitive strains (%)	MIC range [ug/ml]	MIC50	MIC90
CDR	60 (100)	0.16–2	0.19	0.5
ERV (EUCAST)	60 (100)	0.094–2	0.38	1
ERV (FDA)	44 (73)	0.094–0.5	0.38	0.5
PLZ	47 (78)	0.38–1	0.75	0.75
TIG	60 (100)	0.25–1.5	0.5	1.5
FOS (MTS™)^a^	41 (68)	4–32	24	32
FOS (AD FOSF®)^b^	50 (83)	4–32	16	32
CAZ/AVI	0 (0)	–	–	–
AZT	15 (25)	0.094–0.75	0.38	0.5
CAZ/AVI + AZT	60 (100)	0.94–1	0.19	0.5

The interpretation criteria or the susceptibility testing method used are given in brackets. ^a^Determination by the gradient strip method. ^b^Determination by the reference microdilution method in agar using a commercial test AD Fosfomycin 0.25–256 (Liofilchem, Italy).

The statistical analysis compared the MIC values for the tested antibiotics in the three groups of strains (strains originating from bloodstream infections, strains isolated from urinary tract infections, and 20 strains from lower respiratory tract specimens; bronchoalveolar lavage [BAL], tracheal aspirates). Statistically significant lower MIC values for cefiderocol were obtained in the case of strains isolated from lower respiratory tract infections (*p* = 0.002). MIC values for the combination of aztreonam with ceftazidime/avibactam were lower for isolates originating from urinary tract infections (*p* = 0.004). The last group in which statistical significance was demonstrated, is the MIC value for fosfomycin for *K. pneumoniae* NDM isolated from urine samples, determined by the reference method (*p* = 0.014; Table 4).

**Table 4:** Inter-group comparison of assessed variables (MIC value).

Antibiotic	Group I “B” (*n* = 20)			Group II “U” (*n* = 20)			Group III “P” (*n* = 20)			*p* value^*^
	Me	Q1	Q3	Me	Q1	Q3	Me	Q1	Q3	
CDR	0.25	0.19	0.25	0.25	0.19	0.50	0.19^a^	0.09	0.19	**0.002**
ERAV	0.38	0.38	0.44	0.50	0.38	1	0.50	0.38	0.75	0.09
PLZ	0.63	0.50	0.88	0.75	0.75	1	0.75	0.50	128.50	0.36
TIG	0.50	0.44	0.75	0.75	0.63	1	0.50	0.50	0.88	0.06
FOS (MTS™)^a^	32	24	256	32	16	256	24	24	32	0.47
FOS (AD FOSF®)^b^	32	16	64	8^c^	8	24	16	8	32	**0.014**
CAZ/AVI + AZT	0.25	0.16	0.25	0.13^d^	0.13	0.19	0.25	0.19	0.25	**0.004**

Group I “B”, strains isolated from blood; Group II “U”, strains isolated from urine; Group III “P”, strains isolated from bronchoalveolar lavage/tracheal aspirate; Me, median; Q1, first quartile; Q3, third quartile. ^*^Kruskal–Wallis test. ^a^Determination by the gradient strip method. ^b^Determination by the reference microdilution method in agar using a commercial test AD Fosfomycin 0.25–256 (Liofilchem, Italy). ^c^Statistically significantly lower result compared to group I (*post-hoc* test). ^d^The statistically significant lowest result compared to the other two groups (*post-hoc* test). Bold values indicate statistically significant values.

## 4 Discussion

The COVID-19 pandemic witnessed a notable rise in the prevalence of multidrug-resistant strains, especially within the *Enterobacterales* family. An earlier investigation examined bacterial bloodstream infections in patients hospitalized before and during the COVID-19 pandemic (Słabisz et al., 2023) demonstrated a statistically significant increase in the frequency of BSIs caused by NDM-producing *K. pneumoniae*. A report by the European Centre for Disease Prevention and Control (ECDC) published in 2022 (World Health Organization, 2022) on the epidemiological situation in European countries from 2016 to 2020 confirmed the presence of a growing antimicrobial resistance problem among microorganisms, including carbapenem-resistant strains of *K. pneumoniae*. The number of unique cases of CPE strains in Poland from 2019 to 2021, confirmed by the National Reference Center for Antimicrobial Susceptibility Testing of Microorganisms, increased from 2064 to 4,172. In 2019, 1,527 cases of NDM strains were confirmed. In 2021, this number increased to 3,036. These isolates originated only from infection cases and not from intestinal carriages (Hryniewicz et al., 2022). Due to the growing number of patients colonized with CPE strains, not only in the gastrointestinal tract but also in the urinary tract, it is necessary to establish guidelines for empirical treatment and drug susceptibility assessment in patients with suspected MDR strains, including CPE. Single-focal epidemics were frequently observed during the COVID-19 pandemic in the hospital as well as in the post-pandemic period. Patients with rectal colonization of NDM *K. pneumoniae* had a higher risk of bacteriemia than those with KPC *K. pneumoniae* (Pereira et al., 2023).

Our study analyzed the *in vitro* activity of new antibiotics recommended in the IDSA and ESCMID guidelines for treating CPE strains. Currently, the broadest registered antibiotics indicated for the treatment of the source of infection are fosfomycin IV, aztreonam, and ceftazidime in combination with avibactam. Plazomicin and eravacycline have narrow indications for use, with the former registered for the treatment of complicated urinary tract infections and the latter for complicated infections within the abdominal cavity. Cefiderocol, a new broad-spectrum cephalosporin, is also an attractive alternative. Our study assessed the drug susceptibility of 60 strains of NDM-producing *K. pneumoniae*. Due to the high toxicity of colistin and increasing resistance, new antibiotics are useful alternatives in treating infections.

Our study demonstrated the highest sensitivity of 100% for cefiderocol, eravacycline, tigecycline, and a combination of ceftazidime/avibactam with aztreonam. When B-lactamase inhibitors (BLI) are combined with known B-lactams, they demonstrate excellent activity against MBLs. Avibactam forms a stable and hydrolysis-resistant complex with the β-lactamase molecule, causing inhibition of β-lactamases of classes A, B, and partially D (according to Ambler’s classification), including β-lactamases with a highly extended spectrum of activity (KPC and OXA– 38), as well as the chromosomal cephalosporinase AmpC (Behzadi et al., 2020). Due to the narrow indications for using eravacycline, ceftazidime/avibactam and aztreonam are the drugs of choice for bacteremia or pneumonia (Zhanel et al., 2016; Falcone et al., 2021; Sanz Herrero, 2022; Brauncajs et al., 2023). In July 2023, Mark G. Wise reported data on the evaluation of the *in vitro* activity of aztreonam/avibactam, a new antibiotic, against *Enterobacterales* isolates. In total, 24,937 isolates from 27 countries were assessed. Aztreonam/avibactam inhibited 99.1% of CRE isolates (European Committee on Antimicrobial Susceptibility Testing, 2023). The study demonstrated 100% effectiveness of ceftazidime/avibactam in combination with aztreonam. The advantage of this combination of antibiotics is their synergism of action and the ease of determining drug susceptibility using the “strip-stacking” method. The results of this study suggest that most clinical laboratories, using routinely applied methods, can perform the sensitivity determination for combinations of two drugs.

“Strip-stacking” method is relatively easy to perform, fast and shows high correlation with the reference method of microdilution in broth (Khan et al., 2021). In our study, all isolates were sensitive to cefiderocol, but one exhibited borderline susceptibility (MIC = 2 μg/mL). In the latest update of the IDSA guidance, cefiderocol and CAZ/AVI plus AZT are recommended antibiotic for treating NDM-producing *Enterobacterales* with (Tamma et al., 2023).

In the Phase III, open-label study (CREDIBLE-CR), an elevated all-cause mortality rate was demonstrated in patients treated with cefiderocol for infections caused by carbapenem-resistant Gram-negative bacilli compared to patients treated with the best available therapy, which was based on colistin (34% vs. 18%; Bassetti et al., 2021). Mortality difference was recorded for infections of *Acinetobacter* spp. and *Pseudomonas aeruginosa*, and there was no difference in patients with no *Acinetobacter* spp. infection. In light of this evidence, the use of cefiderocol appears to be uncertain compared to therapy based on the combination of CAZ/AVI and AZT. There are still lacking od the clinical data, that would enable the analysis for comparing the effectiveness of treatment between both therapeutic regimens.

Eravacyclin was approved in 2014 by the US FDA and the European Medicines Agency (EMA) to treat complicated intra-abdominal infections (Thakare et al., 2018; Scott, 2019). In Zou et al. (2023) demonstrated the antibacterial activity of eravacycline against CRE. In the study group, 48 strains of *E. coli* were carriers of the NDM gene, and two were KPC. The sensitivity to eravacyclin in this group was 92% (Zou et al., 2023). The susceptibility of the tested NDM-producing *K. pneumoniae* strains to eravacycline varied depending on the interpretation criteria applied. The FDA’s breakpoints are more stringent than those of EUCAST, where epidemiological cut-off values (ECOFF) have been proposed. Based on EUCAST, 100% of the tested strains showed susceptibility to eravacycline. By contrast, when interpreting the results according to FDA criteria, only 73% of the strains could be classified as susceptible to eravacycline.

Irrespective of the interpretation criteria, 100% sensitivity to tigecycline has been demonstrated. In the case of the EUCAST criteria, due to the lack of a breakpoint for *K. pneumoniae*, it was necessary to use the ECOFF value for interpretation, which is the same as the MIC adopted by the FDA. However, tigecycline is a bacteriostatic antibiotic with the primary indication for intra-abdominal, skin, and soft tissue infections. High doses are required for nosocomial pneumonia, with an increased risk of toxicity, according to the latest information from the FDA (FDA Drug Safety Communication, 2017).

FDA approved in 2018 plazomicin, which is an aminoglycoside. Plazomicin is registered for the treatment of infections such as: urinary tract infections, including pyelonephritis, bloodstream infections (BSI), and ventilator-associated pneumonia (VAP; Clark and Burgess, 2020). Plazomicin has a registration for two indications: complicated urinary tract infections in a phase 2 trial and EPIC trial and severe infections caused by CRE (BSI, hospital-acquired pneumonia, and VAP) in the CARE trial (Wagenlehner et al., 2019). Plazomicin received FDA approval with a black box warning for potential aminoglycoside class effects, including nephrotoxicity, ototoxicity, neuromuscular blockade, and risks during pregnancy (U.S. Food and Drug Administration, 2018). The balance between side effects and the benefits of antibiotic therapy with plazomicin underscores the drug’s safety in comparison to traditional aminoglycosides. The reported renal toxicity is comparable to that induced by meropenem. While 3% of patients treated with plazomicin experienced renal function impairment, the renal damage associated with plazomicin is reversible. In a study, approximately 80% of patients demonstrated complete renal function at the discharge visit following treatment (Alfieri et al., 2022). In our study, we had 78% susceptibility to plazomicin.

Patients with severe infections caused by carbapenem-resistant *Enterobacterales* who are susceptible to polymyxins, aminoglycosides, tigecycline, or fosfomycin can use intravenous fosfomycin for combined therapy, as suggested by ESCMID guidelines, or if antibiotics combined with β- lactam inhibitors are not avalible (Paul et al., 2022). This study demonstrated differences in intravenous fosfomycin sensitivity depending on the applied determination method, with 83% sensitivity for the reference method. These data align with reports from the global SENTRY surveillance program, where an 82.6% sensitivity to fosfomycin was shown among *K. pneumoniae* and *E. coli* strains producing carbapenemases (Flamm et al., 2019). In a study by Banerjee et al. (2017), the sensitivity among NDM strains was 92.9%, although it dates back to 2017 and utilized the disk diffusion method. Studies conducted in Poland between 2011 and 2020 revealed a lower sensitivity among carbapenemase-positive strains (55%; Mączyńska et al., 2021).

The critical factor in categorizing a strain into a specific sensitivity category is choosing the appropriate determination method. According to the recommendations, the quantitative agar dilution method is considered the reference method (European Committee on Antimicrobial Susceptibility Testing, 2023). Its advantages include ease of interpretation and high repeatability of the results. However, challenges lie in the time-consuming preparation of substrates, the potential for inaccurate drug dilution, and antibiotic inactivation due to high temperatures. Performing the determination using automated systems (BD Phoenix) or E-tests with a gradient diffusion method can result in distortion. One of the reasons for in the absence of comprehensive data on the level of resistance of strains to intravenous fosfomycin in relation to the local epidemiological situation, is the necessity for a simple and reliable method (Kowalska-Krochmal et al., 2022). Commercial kits (AD Fosfomycin 0.25–256, Liofilchem, Italy) significantly facilitate interpretation and leave no doubt about the sensitivity or resistance of the tested strain (Figure 2). In this study, a commercial test (AD Fosfomycin 0.25–256, Liofilchem, Italy) caused a change in the sensitivity category from resistant to sensitive for 12 strains. This undoubtedly resulted from the difficulty of interpreting the determination using the gradient diffusion method, in which numerous growth increments in the zone of inhibition make it impossible to read the MIC value unambiguously and instead lead to its overestimation. Therefore, using a commercial test is advantageous for fosfomycin, enabling its more frequent consideration in treating CPE infections. Statistically lower MIC values for fosfomycin obtained using the reference method confirm the necessity of employing the agar microdilution method to avoid false results. Similar findings have been presented in other literature reports (Croughs et al., 2022; Pereira et al., 2023).

### 4.1 Limitations of the study

The study was conducted in a single center and was based on *in vitro* evidence of antimicrobial activity, meaning that the effects of application on humans were not determined. The reported effects have not been confirmed in humans, and the number of cases and specimens is not representative of the entire population, which indicates the need for further studies.

## 5 Conclusion

The best therapeutic option for treating infections caused by carbapenemase-producing strains seems to be a combination of ceftazidime/avibactam with aztreonam. Due to the safety of using both drugs, cost effectiveness, and the broadest indications for use among the tested antibiotics, this therapy should be the first-line treatment for CPE infections. The second option, with 100% sensitivity of the tested strains, is cefiderocol, but it remains expensive with limited availability. Despite its high *in vitro* sensitivity, eravacycline has limited use due to narrow indications and is restricted only to complicated intra-abdominal infections. The finding of fosfomycin-resistant NDM-positive *K. pneumoniae* confirms the need to perform drug susceptibility testing using the reference agar microdilution method before implementing intravenous fosfomycin therapy. Nevertheless, a comprehensive evaluation of the efficacy of treating infections caused by NDM-producing *K. pneumoniae* strains should include not only *in vitro* susceptibility assessment but also an analysis of clinical cases.

## Data availability statement

The raw data supporting the conclusions of this article will be made available by the authors, without undue reservation.

## Ethics statement

The studies involving humans were approved by the Bioethics Committee of the Lower Silesian Medical Chamber, Poland (approval no. 2/BNR/2023). The studies were conducted in accordance with the local legislation and institutional requirements. The participants provided their written informed consent to participate in this study.

## Author contributions

NS: Conceptualization, Data curation, Formal analysis, Funding acquisition, Investigation, Methodology, Project administration, Software, Visualization, Writing – original draft. PL: Conceptualization, Data curation, Formal analysis, Funding acquisition, Investigation, Software, Visualization, Writing – original draft. JJ: Conceptualization, Data curation, Formal analysis, Funding acquisition, Investigation, Software, Visualization, Writing – original draft. MF: Conceptualization, Data curation, Software, Writing – review & editing. MB: Formal analysis, Supervision, Writing – review & editing. RD-W: Formal analysis, Supervision, Writing – review & editing. UN: Formal analysis, Supervision, Writing – review & editing.
