# Correction: Evaluation of the *in vitro* susceptibility of clinical isolates of NDM-producing *Klebsiella pneumoniae* to new antibiotics included in a treatment regimen for infections

**DOI:** 10.3389/fmicb.2025.1741710

**Published:** 2025-12-31

**Authors:** Natalia Słabisz, Patrycja Leśnik, Jarosław Janc, Miłosz Fidut, Marzenna Bartoszewicz, Ruth Dudek-Wicher, Urszula Nawrot

**Affiliations:** 1 Department of Laboratory Diagnostic, 4th Military Clinical Hospital, Wroclaw, Poland; 2 Department of Microbiology, Wroclaw Medical University, Wroclaw, Poland; 3 Department of Anaesthesiology and Intensive Therapy, Hospital of Ministry of the Interior and Administration, Wroclaw, Poland; 4 Department of Cardiology, 4th Military Clinical Hospital, Wroclaw, Poland; 5 Department of Pharmaceutical Microbiology and Parasitology, Wroclaw Medical University, Wroclaw, Poland

**Keywords:** *Klebsiella pneumoniae*, metallo-beta-lactamase, NDM, susceptibility, aztreonam, eravacycline, fosfomycin, tigecycline

In the published version, the **Funding** statement reads:

“The author(s) declare that no financial support was received for the research, authorship, and/or publication of this article.”

This information is incomplete. The correct Funding statement should be:

“The author(s) declared that financial support was received for this work and/or its publication. Publication of this article was supported by Wroclaw Medical University statutory grant number SUBZ.D230.24.001.”

The original article has been updated.

