# Supplementary material for: Evaluation of the in vitro susceptibility of clinical isolates of NDM-producing Klebsiella pneumoniae to new antibiotics included in a treatment regimen for infections
Source: Front Microbiol. 2024 Apr 5;15:1331628. doi: 10.3389/fmicb.2024.1331628 (PMC11027895; doi:10.3389/fmicb.2024.1331628)
Supplement: Supplementary file 1 [file Table_1.DOCX]

**Supplementary Table 1.**

| **Strain No.^*^** | **CDR** | | **ERAV** | | | **PLZ** | | **TIG** | | **FOS (MTS^TM^)** | | **AD FOSF 0.25-256** | | **CAZ/AVI** | | **AZT** | | **(CAZ/AVI+AZT)**  **strip-stacking** | |
| --- | --- | --- | --- | --- | --- | --- | --- | --- | --- | --- | --- | --- | --- | --- | --- | --- | --- | --- | --- |
|  | **MIC** | **EUCAST** | **MIC** | **EUCAST** | **FDA** | **MIC** | **CLSI** | **MIC** | **EUCAST** | **MIC** | **EUCAST** | **MIC** | **EUCAST** | **MIC** | **EUCAST** | **MIC** | **EUCAST** | **MIC** | **CLSI** |
| 1B | 0.25 | S | 0.38 | S | S | 0.75 | S | 0.38 | S | 16 | S | 8 | S | 256 | R | 256 | R | 0.25 | S |
| 2B | 0.016 | S | 0.38 | S | S | 0.75 | S | 0.38 | S | 16 | S | 16 | S | 256 | R | 0.25 | S | 0.25 | S |
| 3B | 0.19 | S | 0.094 | S | S | 0.75 | S | 0.5 | S | 24 | S | 8 | S | 256 | R | 256 | R | 0.125 | S |
| 4B | 0.19 | S | 0.38 | S | S | 0.5 | S | 0.5 | S | 256 | R | 16 | S | 256 | R | 256 | R | 0.125 | S |
| 5B | 0.25 | S | 0.38 | S | S | 0.5 | S | 0.5 | S | 24 | S | 32 | S | 256 | R | 256 | R | 0.25 | S |
| 6B | 0.19 | S | 0.38 | S | S | 0.5 | S | 0.5 | S | 48 | R | 8 | S | 256 | R | 256 | R | 0.25 | S |
| 7B | 0.38 | S | 0.5 | S | S | 0.5 | S | 0.75 | S | 256 | R | 256 | R | 256 | R | 256 | R | 0.5 | S |
| 8B | 0.19 | S | 0.38 | S | S | 0.38 | S | 0.5 | S | 32 | S | 32 | S | 256 | R | 256 | R | 0.38 | S |
| 9B | 0.25 | S | 0.38 | S | S | 0.75 | S | 0.5 | S | 16 | S | 16 | S | 256 | R | 256 | R | 0.25 | S |
| 10B | 0.38 | S | 1.5 | S | - | 256 | R | 1.5 | S | 256 | R | 64 | R | 256 | R | 0.75 | S | 1.0 | S |
| 11B | 0.25 | S | 1 | S | - | 256 | R | 1 | S | 64 | R | 32 | S | 256 | R | 0.5 | S | 0.19 | S |
| 12B | 0.25 | S | 0.25 | S | S | 0.5 | S | 0.38 | S | 32 | S | 32 | S | 256 | R | 256 | R | 0.125 | S |
| 13B | 0.25 | S | 0.25 | S | S | 0.38 | S | 0.38 | S | 24 | S | 16 | S | 256 | R | 256 | R | 0.19 | S |
| 14B | 0.25 | S | 0.25 | S | S | 0.38 | S | 0.5 | S | 24 | S | 16 | S | 256 | R | 256 | R | 0.125 | S |
| 15B | 0.38 | S | 0.75 | S | - | 0.75 | S | 0.75 | S | 256 | R | 128 | R | 256 | R | 256 | R | 0.75 | S |
| 16B | 0.25 | S | 0.38 | S | S | 256 | R | 0.38 | S | 48 | R | 32 | S | 256 | R | 0.5 | S | 0.25 | S |
| 17B | 0.19 | S | 0.38 | S | S | 0.5 | S | 0.5 | S | 256 | R | 64 | R | 256 | R | 256 | R | 0.25 | S |
| 18B | 0.19 | S | 0.38 | S | S | 0.5 | S | 0.5 | S | 32 | S | 128 | R | 256 | R | 256 | R | 0.125 | S |
| 19B | 0.25 | S | 0.38 | S | S | 1 | S | 0.75 | S | 16 | S | 64 | R | 256 | R | 256 | R | 0.19 | S |
| 20B | 0.5 | S | 1 | S | - | 256 | R | 1.5 | S | 256 | R | 32 | S | 256 | R | 0.5 | S | 0.25 | S |
| 1U | 0.25 | S | 0.5 | S | S | 0.5 | S | 0.75 | S | 32 | S | 8 | S | 256 | R | 256 | R | 0.125 | S |
| 2U | 0.25 | S | 0.38 | S | S | 0.5 | S | 0.75 | S | 32 | S | 8 | S | 256 | R | 256 | R | 0.125 | S |
| 3U | 0.19 | S | 0.38 | S | S | 0.38 | S | 0.75 | S | 32 | S | 8 | S | 256 | R | 256 | R | 0.125 | S |
| 4U | 0.19 | S | 0.5 | S | S | 0.75 | S | 0.5 | S | 24 | S | 8 | S | 256 | R | 256 | R | 0.094 | S |
| 5U | 0.19 | S | 1.0 | S | - | 0.75 | S | 0.5 | S | 256 | R | 32 | S | 256 | R | 256 | R | 0.19 | S |
| 6U | 0.38 | S | 1.0 | S | - | 0.75 | S | 1 | S | 32 | S | 8 | S | 256 | R | 256 | R | 0.125 | S |
| 7U | 0.25 | S | 0.75 | S | - | 0.75 | S | 0.75 | S | 256 | R | 16 | S | 256 | R | 256 | R | 0.125 | S |
| 8U | 2 | S | 0.38 | S | S | 0.75 | S | 0.5 | S | 16 | S | 4 | S | 256 | R | 256 | R | 0.094 | S |
| 9U | 0.5 | S | 1.0 | S | - | 256 | R | 1.5 | S | 256 | R | 32 | S | 256 | R | 256 | R | 0.38 | S |
| 10U | 0.125 | S | 1 | S | - | 256 | R | 1.5 | S | 256 | R | 64 | S | 256 | R | 256 | R | 0.5 | S |
| 11U | 0.38 | S | 0.38 | S | S | 0.75 | S | 0.75 | S | 16 | S | 8 | S | 256 | R | 256 | R | 0.19 | S |
| 12U | 0.75 | S | 0.094 | S | S | 0.75 | S | 0.25 | S | 16 | S | 8 | S | 256 | R | 0.19 | S | 0.125 | S |
| 13U | 0.125 | S | 0.5 | S | S | 0.75 | S | 0.75 | S | 32 | S | 8 | S | 256 | R | 256 | R | 0.125 | S |
| 14U | 0.5 | S | 2 | S | - | 256 | R | 1.5 | S | 16 | S | 16 | S | 256 | R | 0.25 | S | 0.19 | S |
| 15U | 0.125 | S | 0.38 | S | S | 0.75 | S | 0.75 | S | 16 | S | 16 | S | 256 | R | 0.19 | S | 0.125 | S |
| 16U | 0.25 | S | 0.5 | S | S | 1 | S | 0.75 | S | 24 | S | 8 | S | 256 | R | 0.19 | S | 0.094 | S |
| 17U | 1.0 | S | 0.5 | S | S | 1 | S | 1 | S | 256 | R | 256 | R | 256 | R | 256 | R | 0.25 | S |
| 18U | 0.19 | S | 0.38 | S | S | 0.75 | S | 0.75 | S | 24 | S | 8 | S | 256 | R | 256 | R | 0.125 | S |
| 19U | 0.5 | S | 0.094 | S | S | 0.5 | S | 0.25 | S | 16 | S | 8 | S | 256 | R | 0.094 | S | 0.125 | S |
| 20U | 0.25 | S | 1 | S | - | 256 | R | 1.5 | S | 256 | R | 32 | S | 256 | R | 256 | R | 0.125 | S |
| 1P | 0.25 | S | 0.5 | S | S | 256 | R | 0.5 | S | 256 | R | 256 | R | 256 | R | 256 | R | 0.125 | S |
| 2P | 0.25 | S | 0.5 | S | S | 0.5 | S | 0.75 | S | 24 | S | 16 | S | 256 | R | 256 | R | 0.094 | S |
| 3P | 0.19 | S | 0.38 | S | S | 0.5 | S | 0.5 | S | 24 | S | 16 | S | 256 | R | 256 | R | 0.094 | S |
| 4P | 0.19 | S | 0.38 | S | S | 0.5 | S | 0.5 | S | 24 | S | 16 | S | 256 | R | 256 | R | 0.25 | S |
| 5P | 0.19 | S | 0.5 | S | S | 0.75 | S | 1 | S | 32 | S | 16 | S | 256 | R | 256 | R | 0.25 | S |
| 6P | 0.125 | S | 0.38 | S | S | 0.5 | S | 0.38 | S | 24 | S | 16 | S | 256 | R | 256 | R | 0.25 | S |
| 7P | 0.19 | S | 0.38 | S | S | 0.75 | S | 0.5 | S | 24 | S | 32 | S | 256 | R | 256 | R | 0.25 | S |
| 8P | 0.5 | S | 2 | S | - | 256 | R | 1 | S | 256 | R | 32 | S | 256 | R | 0.38 | S | 0.5 | S |
| 9P | 0.19 | S | 0.38 | S | S | 0.5 | S | 0.5 | S | 32 | S | 16 | S | 256 | R | 256 | R | 0.19 | S |
| 10P | 0.19 | S | 1 | S | - | 0.5 | S | 0.5 | S | 48 | R | 16 | S | 256 | R | 256 | R | 0.19 | S |
| 11P | 0.19 | S | 0.38 | S | S | 0.5 | S | 0.5 | S | 256 | R | 256 | R | 256 | R | 256 | R | 0.38 | S |
| 12P | 0.19 | S | 0.38 | S | S | 0.5 | S | 0.5 | S | 32 | S | 8 | S | 256 | R | 256 | R | 0.25 | S |
| 13P | 0.25 | S | 1 | S | - | 256 | R | 1.5 | S | 32 | S | 32 | S | 256 | R | 0.5 | S | 0.5 | S |
| 14P | 0.094 | S | 1 | S | - | 256 | R | 1.5 | S | 32 | S | 32 | S | 256 | R | 0.38 | S | 0.25 | S |
| 15P | 0.125 | S | 1.5 | S | - | 256 | R | 1.5 | S | 24 | S | 32 | S | 256 | R | 0.5 | S | 0.5 | S |
| 16P | 0.094 | S | 0.5 | S | S | 0.75 | S | 0.75 | S | 24 | S | 8 | S | 256 | R | 0.19 | S | 0.125 | S |
| 17P | 0.094 | S | 0.38 | S | S | 0.75 | S | 0.75 | S | 4 | S | 8 | S | 256 | R | 256 | R | 0.25 | S |
| 18P | 0.094 | S | 0.38 | S | S | 1 | S | 0.5 | S | 12 | S | 8 | S | 256 | R | 256 | R | 0.25 | S |
| 19P | 0.094 | S | 0.5 | S | S | 0.75 | S | 0.5 | S | 6 | S | 8 | S | 256 | R | 256 | R | 0.19 | S |
| 20P | 0.094 | S | 0.5 | S | S | 0.5 | S | 0.5 | S | 16 | S | 8 | S | 256 | R | 256 | R | 0.19 | S |

^*^ Strain identifier composed of a sequential number and a material symbol, with 'B' for blood, 'U' for urine, and 'P' for bronchoalveolar lavage fluid/tracheal aspirate.
